# Body surface area formulae: an alarming ambiguity

**DOI:** 10.1038/srep27966

**Published:** 2016-06-21

**Authors:** Grzegorz Redlarski, Aleksander Palkowski, Marek Krawczuk

**Affiliations:** 1 Gdansk University of Technology, Department of Mechatronics and High Voltage Engineering, ul. G. Narutowicza 11/12, Gdansk, 80-233, Poland

## Abstract

Body surface area (BSA) plays a key role in several medical fields, including cancer chemotherapy, transplantology, burn treatment and toxicology. BSA is often a major factor in the determination of the course of treatment and drug dosage. A series of formulae to simplify the process have been developed. Because easy-to-identify, yet general, body coefficient results of those formulae vary considerably, the question arises as to whether the choice of a particular formula is valid and safe for patients. Here we show that discrepancies between most of the known BSA formulae can reach 0.5 m^2^ for the standard adult physique. Although many previous studies have demonstrated that certain BSA formulae provide an almost exact fit with the patients examined, all of these studies have been performed on a limited and isolated group of people. Our analysis presents a broader perspective, considering 25 BSA formulae. The analysis revealed that the choice of a particular formula is a difficult task. Differences among calculations made by the formulae are so great that, in certain cases, they may considerably affect patients’ mortality, especially for people with an abnormal physique or for children.

An interest in the calculation of the body surface area (BSA) of humans dates back to 1879, when Meeh formulated the first available BSA formula^1^. Not long after, the DuBois brothers published their version of the formula^2^ introducing height as a variable; that formula has remained a standard until now.

The estimation of the model coefficients appeared to be a key problem. In subsequent years of the twentieth and twenty-first centuries, many studies have tried to identify more precise values for the coefficients of the DuBois formula to accurately describe the human BSA. The most recent studies have used 3D laser scanning techniques to determine the human BSA with a substantially higher number of scanned subjects (3951 in Yu, Lo, Chiou^3^, 188 in Schlich^4^, and 270 in Yu, Lin, Yang^5^). As a result, several formulae for the calculation of the BSA for adults have been developed over the past century (Table 1).

At present the BSA calculations are used in several medical fields. The BSA is one of the basic variables that supports the process of transplantation. On the basis of the values of the BSA calculated by the Mosteller formula, the total liver volume index is determined^6^. According to other sources^7^, the BSA is also appropriate as a measure of the dosage of lamivudine in the treatment of chronic hepatitis B.

A fast and accurate determination of the BSA and TBSA (total body surface area, which represents a burned part of the body) is essential during the treatment of burns and is used to predict patients’ chances of survival^8^,^9^. The BSA is necessary to determine the dose of drugs to be administered and, in later stages of treatment, is used to assess the surface of the skin required for transplantation.

The BSA can also be used to calculate the glomerular filtration rate (GFR)^10^,^11^, which represents the amount of filtered plasma per unit of time into the primary urine by the renal glomerulus. Therefore, this factor allows the assessment of the degree of renal function, although its widespread determination on the basis of the BSA is questionable because of the inaccuracy of estimation and, thus, its poor correlation with GFR, especially in children and obese and anorexic individuals. Nevertheless, the BSA remains a major variable in the treatment of nephrotic syndrome^12^.

In most medical cases, the DuBois formula is most commonly used^13^. The latest sources^14^,^15^,^16^ documenting the validity of the calculation of drug doses for chemotherapy based on the BSA have emphasised its most important benefit, which is an increased patient survival rate. The need to rely on the BSA in the near future is also obvious. Nevertheless, there is also a well-known series of studies that have reported on severe consequences resulting from the incorrect use of the BSA as a measure for drug dosing^16^,^17^,^18^,^19^. Strong scientific evidence of its limitations has been provided, followed by a statement that the need to develop appropriate procedures will be one of the most significant challenges in the nearest future.

Likewise, Gurney^20^ has stated that the reliance on an erroneous value of the BSA often leads to serious consequences. Underdosing, accounting for up to 30% of patients, has been specified as being particularly adverse. Weak anti-tumour activity can contribute to up to a 20% reduction in patients’ survival rates. Gurney has also documented that the use of the BSA requires the development of new methods, especially because the potential need for its use in the coming decades.

The BSA will continue to be widely used in the selection of doses for cytotoxic chemotherapy^21^, for which the erroneous application of the BSA leads to changes in the rate of metabolism or kidney and liver dysfunctions, which may affect up to 30% of patients. Certain scientists have also drawn attention to the importance of the BSA as a variable in the calculation of the treatment costs–e.g., by the proper selection of the size of vials of single-use, expensive drugs^21^.

Moreover, several antivirals^22^, antimicrobials^23^ and antifungals^24^ require a dosing regimen based on the BSA. It is thought that drugs, which accumulate in the extracellular fluid, should be dosed on the basis of the BSA because it reflects the volume of extracellular fluid and the total volume of body water more precisely than the body weight itself^25^. The BSA is also an indicator associated with skin water evaporation^26^ and an indicator for several problems associated with the assessment of environmental occupational risk.

BSA scaling has been recommended by the U.S. Food and Drug Administration as a method for using data from animal model species to establish safe starting dosages for initial human clinical trials^27^. The widespread use of the BSA formulae, mainly those based on the Meeh formula, in veterinary medicine confirms its wide scope of applications. However, even scientists from this field have started to consider current formulae as being inaccurate because they were established with limited data^28^.

## Results

**Figure 1:**
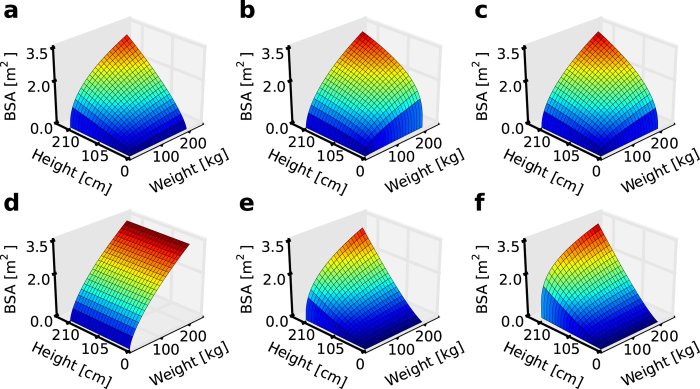
Examples of BSA surfaces in accordance with various formulae. (**a**) DuBois & DuBois. (**b**) Boyd. (**c**) Gehan & George. (**d**) Livingston & Lee. (**e**) Schlich. (**f**) Nwoye.

All recognised BSA formulae are based on easy-to-identify body variables, such as height, weight, and, in certain cases, the age and sex of the patients. However, the formulae differ in a meaningful range (Fig. 1). Hence, in clinical practice, the choice of formula used depends only on the attending doctors who often, despite their knowledge and experience, cannot identify errors between the sets of formulae available. Variability among individual patients may corrupt the results obtained because all BSA formulae were established on the basis of an isolated, standard-physique adult group of people. None of the current formulae consider such invariability–e.g., non-standard body proportions, prominent and massive bone structure, and dwarfism. The consequences of the errors caused by the BSA miscalculation can be severe. Using too-small doses of drugs during chemotherapy may result in the recurrence of the cancer, whereas using doses that are too high during the treatment may lead to serious side effects or even death. There are also the risks of organ transplantation failure due to incorrect BSA estimation.

**Figure 2:**
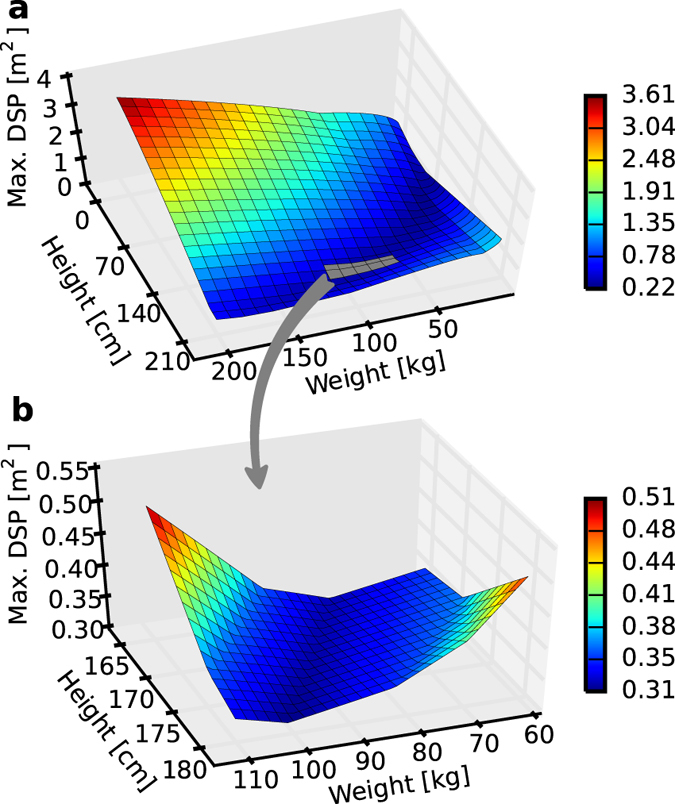
Maximum discrepancy between any two of the 25 BSA estimation methods. (**a**) For a full scope of up to 200 kg in weight and 210 cm in height. (**b**) For a limited scope of 60–110 kg in weight and 165–180 cm in height (standard adult physique).

The most significant differences between the BSA values are documented in Fig. 2. The obtained discrepancies concerned a comparative analysis relating to the calculation of the BSA for men. Figure 2 shows the highest difference between any two of 25 considered BSA formulae (Table 1). The entire scope of possible weight-height combinations is shown in Fig. 2a. Notably, the lowest possible discrepancy was equal to 0.22 m^2^. All of the tested formulae were mostly in agreement with each other in the weight midrange and height upper range because all of the considered BSA formulae were established mostly on the basis of a standard adult physique; therefore, they should provide the lowest possible error for that range. However, the rather large discrepancy from 0.22 to 1.2 m^2^ was still sufficiently high to disregard any justification of which formula should be considered the most accurate. The extreme BSA differences started at the low height values, which is cause for alarm if BSA calculations made for short and obese adults or children are to be considered. A closer look into a standard-physique range of discrepancies (Fig. 2b) revealed that the lowest noted difference was not even present for that range. The discrepancies varied from 0.31 m^2^ in a line from low weight and height to high weight and height values to 0.51 m^2^ for a high weight and low height and for a low weight and high height.

**Figure 3:**
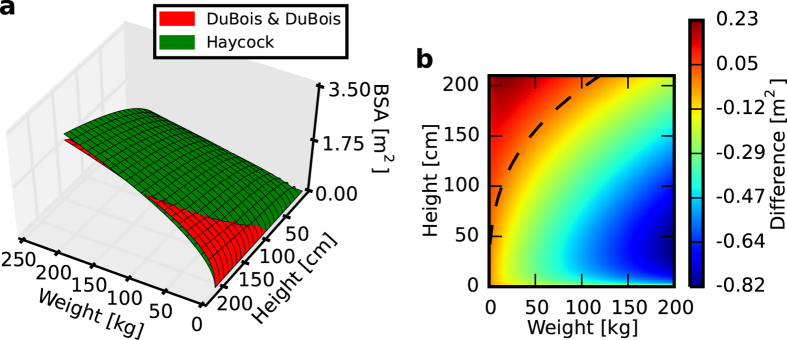
Comparison of the changes in BSA according to DuBois & DuBois and Haycock formulae. (**a**) Intersection of the surfaces resulting from the two formulae. (**b**) Difference between the two formulae. The dashed line indicates equal BSA values.

**Figure 4:**
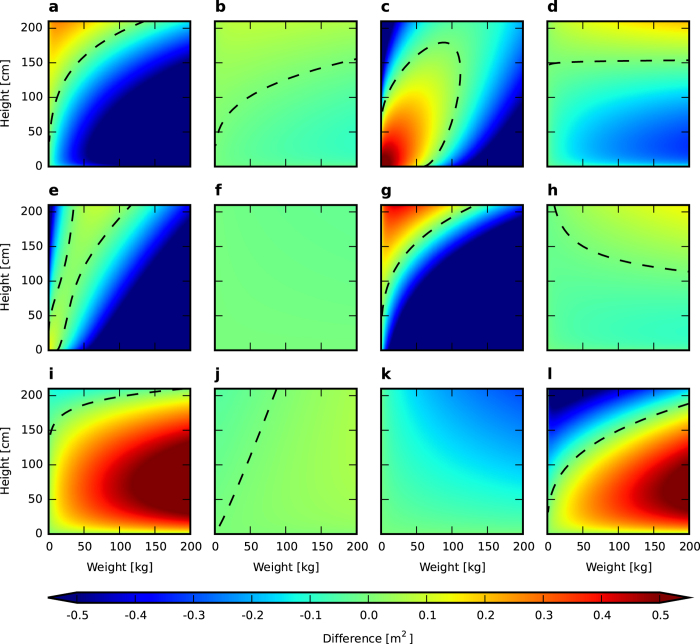
Differences between DuBois & DuBois and an additional 12 other BSA formulae. (**a**) Boyd. (**b**) Fujimoto. (**c**) Mattar. (**d**) Nwoye & Al-Sheri. (**e**) Stevenson. (**f**) Takahira. (**g**) Livingston & Lee. (**h**) Mehra. (**i**) Schlich. (**j**) Yu, Lin, Yang. (**k**) Faber & Melcher. (**l**) Nwoye. The dashed line indicates equal BSA values.

Most of the known BSA formulae are consistent with each other in a very limited range. The points of their consistency form a curve, or a set of curves, across a surface indicated by BSA values. It is particularly apparent when two BSA surfaces are compared with each other (Fig. 3a). In this case, the two surfaces intersect close to the area of low weight and high height values. Differences between the DuBois and Haycock formulae reached up to 0.82 m^2^ for a high weight; however, for a standard adult physique, their maximum was equal to approximately 0.2 m^2^ (Fig. 3b). If one compares the DuBois & DuBois formula–one of the most commonly used formulae–with several other formulae, even more diversity can be observed (Fig. 4). A range of differences was apparent from a match to above 0.5 m^2^ in BSA value. However, it is most interesting to note that the consistency curve was entirely different for each case. There are examples that entirely lacked an intersection, such as with the Takahira and Faber & Melcher formulae (Fig. 4f,k), and others in which the curves differed greatly in shape and placement (e.g., Fig. 4c,e). However, for most examples, the curve was convergent with what is shown in Fig. 3b (which was true for other formulae as well). Because of such diversity between the pairs, it is therefore unclear which one of the compared formulae is most suitable for a specific range of physique.

**Figure 5:**
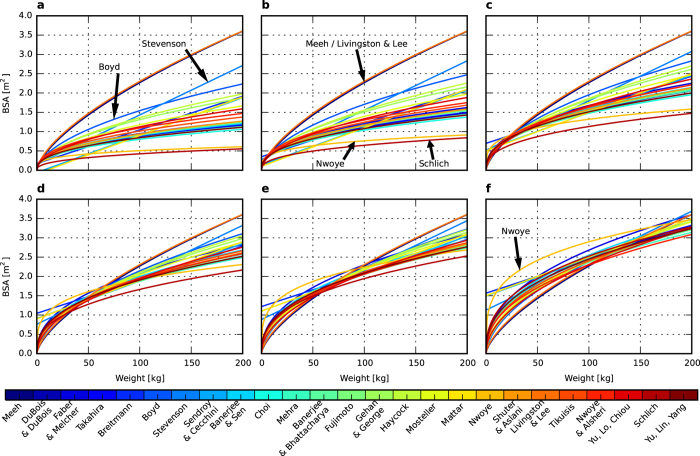
Comparison of the BSA change as a function of weight for all 25 BSA formulae. (**a**) for 50 cm in height. (**b**) for 70 cm in height. (**c**) for 110 cm in height. (**d**) for 150 cm in height. (**e**) for 170 cm in height. (**f**) for 210 cm in height.

To depict how all of the considered BSA formulae influence the results, a collection of images that show a change in BSA as a function of weight for several heights (Supplementary Video 1, Fig. 5) was prepared. The results were consistent with the maximum difference surface (Fig. 2a)–the largest discrepancy occurred for low heights and decreased with the height increase. However, even at the point of the lowest variation (approximately 200 cm Fig. 5f), the differences reached approximately 0.5 m^2^. All of the presented curves shifted in a different grade, as indicated by the progressive amount of change across subsequent heights. The BSA values obtained from certain formulae changed substantially (e.g., for the Nwoye formula), whereas others were characterised by a small variation in successive heights (e.g., for the Boyd formula). Additionally, there are formulae that are independent of height (Meeh and Livingston & Lee) and therefore are unfit for cases of abnormal physique.

To support the claims presented above, a few examples of BSA values should be discussed. Supplementary Fig. 1 presents BSA values according to the 25 formulae in question for a selection of human physique, primarily that of children and anorexic and obese adults.

For an average 6-year-old male child (weight: 20.6 kg; height: 115.5 cm), the BSA ranges from 0.66 m^2^ (according to the Schlich formula) to 0.927 m^2^ (according to the Nwoye formula). With the mean value equal to 0.819 m^2^ and standard deviation equal to 0.051, this example still does not provide any degree of certainty regarding whether any of the formulae is accurate.

The deviation is even higher for examples of abnormal physique. A very severely obese male 6-year-old child (weight: 50 kg; height: 115.5 cm) should have the BSA ranging from 0.925 m^2^ (according to the Schlich formula) to 1.472 m^2^ (according to the Livingston & Lee formula). The mean value for all formulae equals 1.216 m^2^, whereas the standard deviation is 0.111.

The most extreme case presented is of a very severely obese average height male adult (weight: 350 kg; height: 175 cm), for which the BSA ranges from 3.223 m^2^ (according to the Nwoye formula) to 5.395 m^2^ (according to the Stevenson formula) with the mean value of 4.033 m^2^ and standard deviation equal to 0.591.

It should be emphasised that there is no pattern whatsoever regarding whether a certain formula generally yields higher or lower BSA values. Almost all of the formulae deviate substantially depending on the case. This is particularly evident when the percentage deviation from the mean is compared (Supplementary Fig. 2).

Based on the selected examples of BSA calculation presented above, a brief case study of dose calculation can be performed to illustrate the differences. Irinotecan, a known drug used for the treatment of cancer, can be administered (often in combination with cisplatin) as a 90-minutes continuous intravenous infusion at a dose level between 175 and 350 mg/m^2^ 29. For an extreme case of a very severely obese average height male adult (weight: 350 kg; height: 175 cm) the maximum irinotecan dose spans from 1,128.05 to 1,888.25 mg. The difference is higher that the recommended average fixed dose for irinotecan treatment. A less extreme example shows significant differences as well. For an average weight and height male adult (weight: 80 kg; height: 180 cm) the maximum irinotecan dose spans from 668.85 to 793.01 mg.

## Discussion

The BSA as a major factor in several medical procedures should be carefully studied. However, all BSA-related studies so far do not present any viable conclusions whether or which BSA formula is accurate enough for a general medical treatment. Based on a broader than previous studies scale with 25 formulae compared, this paper proves that it is indeed an issue. There are several causes of the meaningful discrepancies among the calculated BSA values:

1. The formulae parameters were estimated independently for subjects from different ethnic groups (e.g., people from the Far East, British people, and Germans).
2. A few of the patients’ variables were considered (height, weight, and, in particular cases, sex and age).
3. There was a lack of additional variables indicative of character variation (abnormal physique as a result of impaired development–e.g., dwarfism, gigantism, and anorexia).
4. The model parameters were estimated by matching to a specific simple function.

Therefore, previous studies have relied on the search for a global optimum. The formulae allow for the highest possible correlation of the BSA ratio with limited state variables for a representative sample of patients, determined by the individuals enrolled therein. This approach, despite the use of accurate measurement techniques and a high accuracy of estimated parameters^3^,^4^,^5^, could not eliminate the above mentioned drawbacks. Based on the presented results, these disparities particularly concern children, individuals from other geographical regions, people with an abnormal physique, or people who are sick or debilitated as a result of disease or drug abuse.

Moreover, it is indicated by several recent publications^17^,^18^ that the BSA may be in fact inappropriate as a measure for drug dosing regimen–especially for chemotherapy drugs. However, having regard to the results presented above, a question can be raised whether those limitations are due to the fact that most studies that raised them were performed on false premises that the formulae used are accurate? It is notable that all articles that deal with BSA calculation and BSA-based dosing review and compare only few formulae, which narrows the perspective. Furthermore, this article proves that chances of BSA miscalculation are substantial and there is no hard evidence that proves whether any of the formulae presented is more appropriate for a specific case other than a limited group of patients based on which it was developed.

Maybe the most important conclusion from the analysis presented in the paper is how BSA estimation based on the formulae presented may impact medical procedures and, ultimately, patients’ chance of survival. Certain medical procedures mentioned are in fact life-saving, thus a clear connection between BSA miscalculation and patients’ death due to misuse of treatment can be drawn. As mentioned in the Introduction, according to studies performed by Gurney^20^ over 30% of patients receiving cytotoxic chemotherapy are affected by unrecognised underdosing (with BSA being the main dosing factor). This in turn leads to, i.a., an almost 20% relative reduction in survival for women receiving adjuvant chemotherapy for breast cancer. Therefore, it is clear that underdosing due to underestimated BSA–and as we show in the paper, it is indeed an issue–may affect patients’ mortality on a significant scale.

To summarise the importance of research in this area, it should be clearly stated that the exact BSA calculation is one of the most important issues in the processes that support the effectiveness of the treatment of numerous diseases, primarily those that, if untreated or treated incorrectly, lead to the death of patients. The currently used formulae for the determination of the BSA, including the latest ones, not only do not allow for the precise determination of the body surface area but also provide no information concerning which one, in relation to the individual variability of patients, leads to the most accurate results. As stated in the introduction, the development of more comprehensive BSA estimation formulae should be a priority for researchers who address the treatment of cancer and other diseases as well as in the other medical fields mentioned.

## Methods

The analysis presented is based entirely on calculations made according to the most widely known BSA formulae (Table 1). Therefore, all of the figures and results presented are a representation of multiple ways of proving the variability of the current BSA formulae. Most results depicted on figures were produced for a range of 0–210 cm of height and 0–200 kg of weight. All calculations were performed on a PC with an Intel Core i7-3770 CPU. The analysis was performed with the use of the Python programming language. Figures were produced with the Matplotlib python library^30^.

Figure 2 shows the maximum difference between any two of the 25 BSA formulae for each combination of weight and height. For each point on the weight-height plane, the smallest BSA value is subtracted from the largest value and represents a part of the surface.

Figures 3 and 4 are the results of intersecting BSA values according to the DuBois & DuBois formula with BSA values from several other formulae. All are presented on a three-dimensional plane as a literal surface intersection (Fig. 3a) and as colourmaps that represent the difference between BSA values according to the DuBois & DuBois formula and BSA values from the other formulae.

Figure 5 presents how the values of BSA according to all formulae in question change for a fixed height. The result is particularly important if considered for adults because their height does not significantly change over time. Therefore, with a change in weight comes a major change in BSA values; however, the nature of the change strongly depends on the height and is inconsistent for all formulae except the Meeh and Livingston & Lee formulae (because they depend only on weight).

## Additional Information

**How to cite this article**: Redlarski, G. *et al*. Body surface area formulae: an alarming ambiguity. *Sci. Rep.* **6**, 27966; doi: 10.1038/srep27966 (2016).

## Supplementary Material

Supplementary Information

Supplementary Video 1

## Figures and Tables

**Table 1:** Body Surface Area formulae used for the comparison.

Authors	Formula	Reference
Meeh (1879)	0.1053 ⋅ *W*^2/3^	1
DuBois & DuBois (1916)	0.007184 ⋅ *W*^0.425^ ⋅ *H*^0.725^	2
Faber & Melcher (1921)	0.00785 ⋅ *W*^0.425^ ⋅ *H*^0.725^	31
Takahira (1925)	0.007246 ⋅ *W*^0.425^ ⋅ *H*^0.725^	32
Breitmann (1932)	0.0087 ⋅ (*W* + *H*) − 0.26	33
Boyd (1935)	0.0003207 ⋅ (W ⋅ 1000)^0.7285 − 0.0188 ⋅ log^_10_^(*W* ⋅ 1000)^ ⋅ *H*^0.3^	34
Stevenson (1937)	0.0128 ⋅ *W* + 0.0061 ⋅ *H *− 0.1529	35
Sendroy & Cecchini (1954)	0.0097 ⋅ (*W* + *H*) − 0.545	36
Banerjee & Sen (1955)	0.007466 ⋅ *W*^0.425^ ⋅ *H*^0.725^	37
Choi (1956)	men: 0.005902 ⋅ *W*^0.407^ ⋅ *H*^0.776^ women: 0.008692 ⋅ *W*^0.442^ ⋅ *H*^0.678^	38
Mehra (1958)	0.01131 ⋅ *W*^0.4092^ ⋅ *H*^0.6468^	39
Banerjee & Bhattacharya (1961)	0.007 ⋅ *W*^0.425^ ⋅ *H*^0.725^	40
Fujimoto *et al*. (1968)	0.008883 ⋅ *W*^0.444^ ⋅ *H*^0.663^	41
Gehan & George (1970)	0.0235 ⋅ *W*^0.51456^ ⋅ *H*^0.42246^	42
Haycock *et al*. (1978)	0.024265 ⋅ *W*^0.5378^ ⋅ *H*^0.3964^	43
Mosteller (1987)	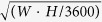	44
Mattar (1989)	(*W* + *H* − 60)/100	45
Nwoye (1989)	0.001315 ⋅ *W*^0.262^ ⋅ *H*^1.2139^	46
Shuter & Aslani (2000)	0.00949 ⋅ *W*^0.441^ ⋅ *H*^0.655^	47
Livingston & Lee (2001)	0.1173 ⋅ *W*^0.6466^	48
Tikuisis (2001)	men: 0.01281 ⋅ *W*^0.44^ ⋅ *H*^0.6^ women: 0.01474 ⋅ *W*^0.47^ ⋅ *H*^0.55^	49
Nwoye & Al-Sheri (2003)	0.02036 ⋅ *W*^0.427^ ⋅ *H*^0.516^	50
Yu, Lo, Chiou (2003)	0.015925 ⋅ (*W* ⋅ *H*)^0.5^	3
Schlich (2010)	men: 0.000579479 ⋅ *W*^0.38^ ⋅ *H*^1.24^ women: 0.000975482 ⋅ *W*^0.46^ ⋅ *H*^1.08^	4
Yu, Lin, Yang (2010)	0.00713989 ⋅ *W*^0.404^ ⋅ *H*^0.7437^	5

*W* indicates weight in kilograms, and *H* indicates height in centimetres.
