# Supplementary material for: Body surface area formulae: an alarming ambiguity
Source: Sci Rep. 2016 Jun 21;6:27966. doi: 10.1038/srep27966 (PMC4914842; doi:10.1038/srep27966)
Supplement: Supplementary Information [file srep27966-s1.pdf]

Body surface area formulae: an alarming ambiguity

Grzegorz Redlarski, Aleksander Palkowski, and Marek Krawczuk

Supplementary Information

Supplementary Figure 1

Title: BSA values for a selection of human measurements. The bars represent BSA values according to all 25 formulae considered. **a**: Normal male 6-years-old child (weight: 20.6 kg; height: 115.5 cm). **b**: Very severely obese male 6-years-old child (weight: 50 kg; height: 115.5 cm). **c**: Average height male adult with extreme anorexia nervosa (weight: 45 kg; height: 175 cm). **d**: Very severely obese average height male adult (weight: 350 kg; height: 175 cm). **e**: Above average height male adult with extreme anorexia nervosa (weight: 50 kg; height: 200 cm). **f**: Very severely obese under average height male adult (weight: 160 kg; height: 130 kg). The red lines indicate mean values.

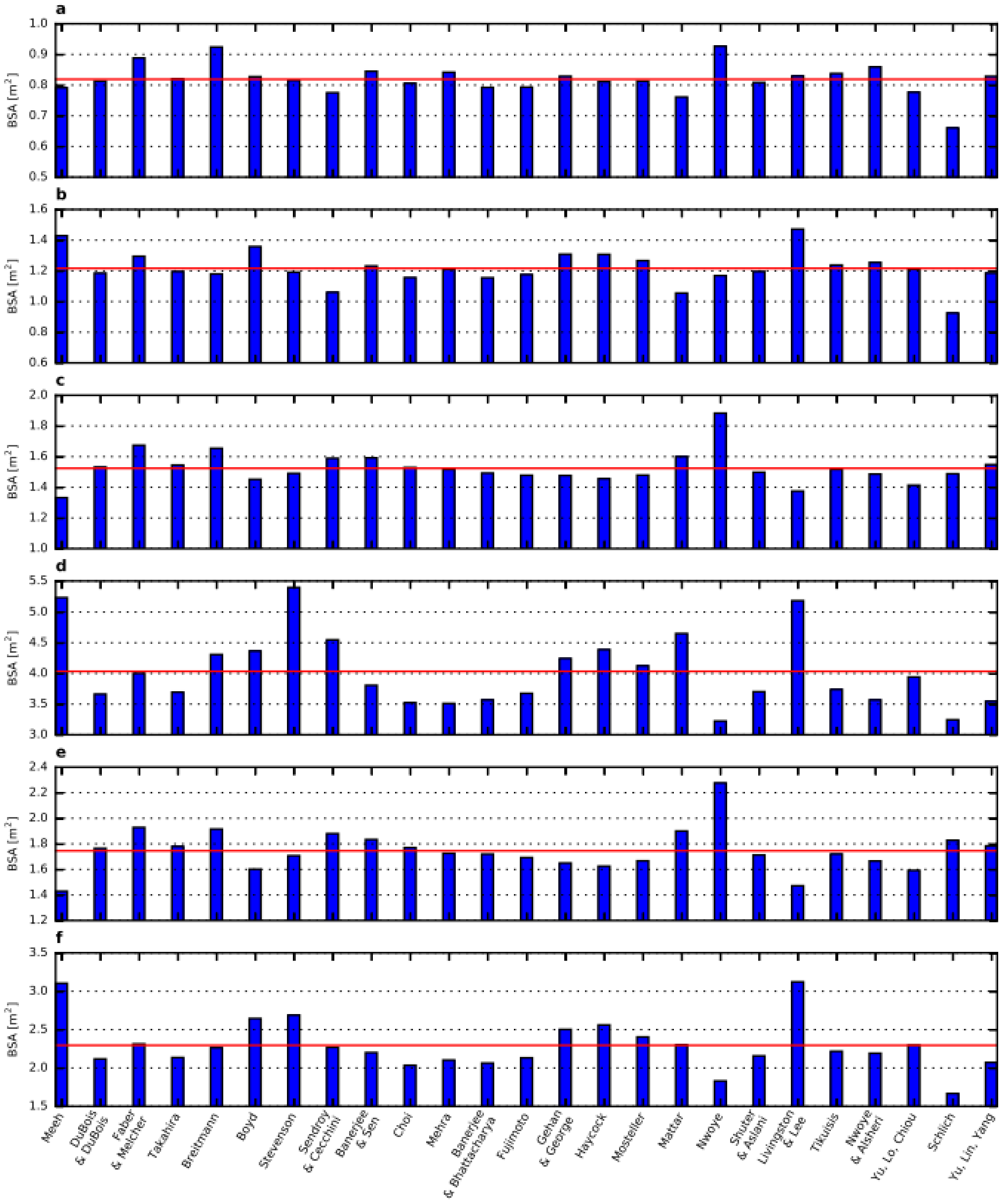

Supplementary Figure 2

Title: Percentage deviation from the mean of BSA values for a selection of human measurements. The bars represent a deviation from the mean according to Supplementary Fig. 1. Values above the mean are indicated in blue, whereas those under the mean—in green. **a**: Normal male 6-years-old child (weight: 20.6 kg; height: 115.5 cm). **b**: Very severely obese male 6-years-old child (weight: 50 kg; height: 115.5 cm). **c**: Average height male adult with extreme anorexia nervosa (weight: 45 kg; height: 175 cm). **d**: Very severely obese average height male adult (weight: 350 kg; height: 175 cm). **e**: Above average height male adult with extreme anorexia nervosa (weight: 50 kg; height: 200 cm). **f**: Very severely obese under average height male adult (weight: 160 kg; height: 130 kg).

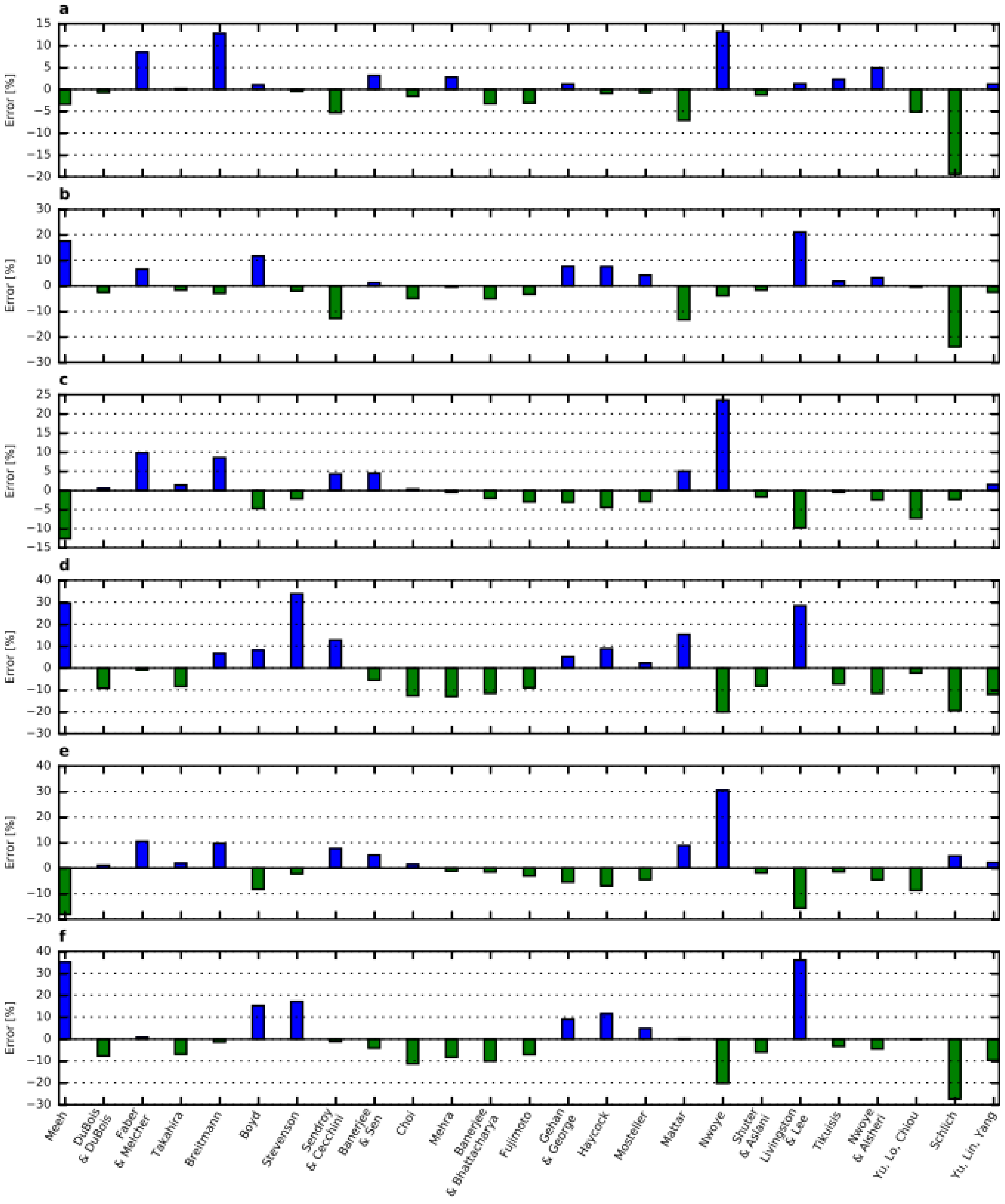

Supplementary Video 1

File: BSA_change_animation.avi
Title: Collective comparison of BSA change.
Legend: The animation presents a fluent change in function of weight for all 25 BSA formulae considered (Table 1).
